# Outcome of scleral buckling with or without gas tamponade for recurrent retinal detachment in post-vitrectomy eyes

**DOI:** 10.1186/s12886-021-01873-y

**Published:** 2021-02-27

**Authors:** Jun-Xing Bai, Xiao-Jian Zhang, An-Li Duan, Xiao-Yan Peng

**Affiliations:** 1 Beijing Institute of Ophthalmology, Beijing Tongren Eye Center, Beijing Tongren Hospital, Capital Medical University, Beijing Ophthalmolgy and Visual Science Key Laboratory, No.17 Hougou Lane, Chongnei Street, Beijing, 100005 People’s Republic of China; 2 Department of Ophthalmology, Beijing Meiermu Hospital, No.65 Fuxing Road, Haidian District, Beijing, 100036 People’s Republic of China; 3 Department of Ophthalmology, Beijing Huade Eye Hospital, No.179 North Chaoyang Road, Chaoyang District, Beijing, 100101 People’s Republic of China; 4 Beijing Tongren Eye Center, Beijing Tongren Hospital, Capital Medical University, Beijing Ophthalmolgy and Visual Science Key Laboratory, No.1 Dongjiaominxiang, Dongcheng District, Beijing, 100730 People’s Republic of China

**Keywords:** Recurrent retinal detachment, Scleral buckling, After pars plana vitrectomy, Post-vitrectomy eye

## Abstract

**Background:**

Treatment of recurrent retinal detachment (re-RD) following vitrectomy (post-gas/air tamponade and post-silicone oil removal) is challenging. Previously reported treatment is commonly revision pars plana vitrectomy (PPV) combined with tamponade, which is invasive and a burden both economically and emotionally when compared with scleral buckling (SB). The purpose of this study is to report anatomical and functional outcomes of SB with or without gas tamponade in eyes with recurrent retinal detachment (re-RD) that previously underwent PPV at least once.

**Methods:**

We retrospectively reviewed the medical records of 14 patients (14 eyes) who underwent PPV at least once and were treated with SB after re-RD. Preoperative characteristics, intraoperative complications, and postoperative data were assessed. The final anatomical and functional outcomes were analyzed.

**Results:**

The original PPV was performed for primary rhegmatogenous retinal detachment in 11 eyes, macular hole retinal detachment in 2 eyes, and myopic foveoschisis in 1 eye. Previously, 3 eyes underwent one PPV with gas tamponade, and the remaining 11 (79%) eyes underwent 2–5 operations. Seven eyes underwent the procedure with gas injection. At the last follow-up, 13 eyes achieved total retinal attachment and 1 eye had re-RD. The postoperative intraocular pressure was within the normal range, except in 1 eye (6 mmHg). The finest postoperative best-corrected visual acuity (BCVA) was 20/25. There was a significant improvement in BCVA from 20/160 ± 20/63 at baseline to 20/80 ± 20/50 at the last visit in the 13 successfully treated eyes (*P* = 0.025).

**Conclusions:**

SB can be effective for re-RD after PPV in specific cases.

## Background

Treatment of recurrent retinal detachment (re-RD) following vitrectomy (post-gas/air tamponade and post-silicone oil removal) is challenging. Treatment mainly includes scleral buckling (SB) and revision pars plana vitrectomy (PPV) combined with tamponade [1], the latter of which is invasive and a burden both economically and emotionally when compared with SB. The tamponade agent commonly used is silicone oil, and the eyes can easily become oil-dependent. Many studies have reported the outcomes of surgery for re-RD [1–9]; however, there appear to be no clinical trials evaluating the role of SB in treating re-RD in post-vitrectomy eyes. SB creates an area of contact by bringing the retinal pigment epithelium near the retinal neuroepithelium around the break, by the indentation of the scleral wall to close the break, thus relieving and releasing the vitreous traction and supporting the retinal breaks [2, 10]. SB is less traumatic, and the patient can recover rapidly and comfortably without necessarily being in a prone position or only being so for a short time if assisted with gas tamponade. This study aimed to assess the anatomical and functional outcomes of SB for re-RD in eyes that have previously undergone one or more PPV.

## Methods

The study adheres to the tenets of the Declaration of Helsinki. Surgeries were performed after obtaining informed consent from all patients. Data was obtained through a retrospective review of the medical records of patients who underwent SB between November 2016 and November 2019 to treat re-RD after PPV. Only eyes previously diagnosed with rhegmatogenous retinal detachment (RRD) (including macular hole retinal detachment; MHRD) or myopic foveoschisis that underwent PPV at least once were included. Exclusion criteria were secondary RRD resulting from trauma, endophthalmitis or diabetic retinopathy; re-RD with silicone oil in situ; re-RD due to macular hole; re-RD with posterior proliferative vitreoretinopathy (PVR) or choroidal detachment; and funnel-shaped re-RD. Fourteen consecutive eyes were included.

All eyes had undergone a complete ocular examination, including slit-lamp examination and binocular indirect ophthalmoscopy. The preoperative data collected included the patients’ age and sex, laterality of the eye, lens status, past surgery history, macular status (attached or detached), number and location of breaks, location and extent of re-RD (total or partial in clock hours), PVR grading(Retina Society Terminology Committee Classification 1983 [11]),axial length, best-corrected visual acuity (BCVA) at baseline, intraocular pressure (IOP) by noncontact tonometry, and duration of re-RD. Surgery details during this time and intraoperative complications were recorded. Postoperative data included length of follow-up, BCVA, IOP, retina status, postoperative complications, and additional surgical procedures.

### Surgical techniques

The surgical procedures were performed under local or general anesthesia by the same surgeon (Dr. Duan). Of the 14 patients, 6 eyes underwent primary retinal repair surgery by Dr. Duan and 8 were referred to her for further management after undergoing one or more unsuccessful surgeries by other surgeons (see Table 1). Indirect ophthalmoscopy was used to locate and freeze the retinal breaks. For eyes with more subretinal fluid which influences cryopexy of the breaks, sclerotomy and choroid puncture fluid drainage was performed. For eyes complicated with fluid drainage, an intravitreous injection of 0.9% normal saline was given and/or tightening of the encircling band was performed during the drainage procedure to prevent hypotony. A silicon band or/and silicon tire was used. If necessary, the procedure was performed in combination with a vitreous cavity gas injection. Patients whose eyes were injected with gas were asked to maintain a prone position on the postoperative day, and the position was changed according to the absorption of gas and subretinal fluid.

**Table 1:** Characteristics of all patients in the study

No.	Age	Sex	Eye	AL (mm)	Symptoms duration	RD extent (quadrant)	PVR grading	Break number	Break location	Macular involvement	Lens status	Previous diagnosis	Previous ocular surgery
1	71	M	R	23.76	1 day	2	B	suspicious	inferior	Y	Pseudophakic	MHRD	1. PPV + ILM peeling+silicon oil injection 2. P + I + silicon oil removal
2^a^	46	F	L	26.5	7 days	1	B	1	2 o’clock	N	Phakic	RRD	1. PPV + laser+silicon oil injection 2. silicon oil removal
3	29	M	R	–	15 days	3	B	1	1 o’clock	N	Phakic	RRD	1. PPV + laser+silicon oil injection 2. silicon oil removal
4	40	M	R	24.69	2 days	2	C1	suspicious	inferote-mporal	Y	Pseudophakic	RRD	1. PPV + P + I + laser+air tamponade
5^b^	40	M	R	30.96	2 months	1	B	1	2 o’clock	N	Pseudophakic	RRD	1. Scleral buckling 2. Encircling band removal+P + I + laser+air tamponade
6^d^	69	M	R	–	1 month	4	C1	0	–	Y	Pseudophakic	RRD	1. PPV + P + I + laser+gas tamponade 2. PPV + laser+silicon oil injection 3. Silicon oil removal
7	54	M	R	23.77	1 month	1	B	1	6 o’clock	N	Pseudophakic	RRD	1. P + I 2. PPV + cryo+laser+air tamponade
8	60	M	L	22.44	15 days	2	B	suspicious	inferote-mporal	Y	Pseudophakic	RRD MH^*^	1. Scleral buckling+exoplant+cryo+PPV + gas tamponade 2. P + I 3. PPV + laser+silicon oil injection 4. Silicon oil removal 5. PPV + ILM peeling+C2F6 tamponade
9	29	M	R	27.56	20 days	2	B	3	4–6 o’clock	N	Phakic	MHRD	1. PPV + ILM peeling+ silicon oil injection 2. Silicon oil removal
10	53	M	R	33.17	14 days	1	A	0	–	N	Aphakic	RRD	1. PPV + P + air tamponade
11^c^	45	M	R	29.11	3 days	3	B	suspicious	inferote-mporal	Y	Pseudophakic	RRD	1. PPV + laser+silicon oil injection 2. Silicon oil removal
12	74	M	R	30.22	7 days	2	B	suspicious	inferote-mporal	N	Pseudophakic	Foveoschisis MHRD^*^	1. PPV + P + I + laser+ILM peeling+C2F6 tamponade 2. Posterior scleral reinforcement 3. PPV + ILM tamping+air tamponade
13^e^	64	M	R	24.21	22 days	4	C1	2	3&11 o’clock	Y	Aphakic	RRD	1. PPV + lensectomy+laser+silicon oil injection 2. Silicon oil removal+retina repair+silicon oil reinjection 3. Silicon oil removal
14	38	F	R	23.91	14 days	4	C1	2	2&8–9 o’clock	Y	Phakic	RRD FEVR	1. PPV + laser+gas tamponade

M: male, F: female, AL: axial length, MHRD: macular hole retinal detachment, ILM:inner limiting membrane, P: phacoemulsification, I:IOL implant, RRD: rhegmatogenous retinal detachment, PPV: pars plana vitrectomy, FEVR: familial exudative vitreoretinopathy

Primary retinal repair surgery was done by Dr. Duan in case1,4,7,10,12 and 14. Primary scleral buckling was done by other surgeon in case 5 and the PPV surgery was done by Dr. Duan. The rests were previously treated by other surgeons

^a^ Lasik surgery history before RRD, P + I surgery after scleral buckling and YAG laser capsulotomy later

^b^ Lasik surgery history before RRD, cornea punctate opacity

^c^ Lasik surgery history before RRD

^d^ posterior capsular opacity, cornea limbus is cloudy

^e^ superior temporal corneal leukoma

^*^ Secondary complication, the reason for last surgery. At the time of SB, macular hole was totally closed after the amending PPV surgery

gas tamponade: If we don’t know the exact gas type, we generally referred to as gas tamponade

### Statistical analysis

The Snellen’s visual acuity was converted to logarithm of the minimum angle of resolution (logMAR) equivalent for analysis. The logMAR denotations for non-numeric visual acuities were: counting fingers = 1.7 logMAR, hand motion = 2.0 logMAR, light perception = 2.3 logMAR, and no light perception = 3.0 logMAR [12]. A *P*-value of less than 0.05 was considered statistically significant. Statistical analysis was performed using the SPSS statistical software (version 26, IBM SPSS statistics). Continuous variables were expressed as mean ± SD, and categorical variables were expressed as individual counts and proportions. Univariate analyses were performed using a paired t-test to determine the association between baseline demographics and outcomes after surgical procedures.

## Results

The preoperative characteristics are summarized in Table 1. Of the 14 patients, 12 were male and 2 were female. Patient age ranged from 29 to 74 years (51 ± 15 years). There were 12 right eyes and 2 left eyes. Lens status included 2 (14%) aphakic eyes, 4 (29%) phakic eyes, and 8 (57%) pseudophakic eyes. The original PPV was performed for primary RRD in 11 eyes, macular hole retinal detachment in 2 eyes, and myopic foveoschisis in 1 eye. Three eyes had undergone Lasik surgery before the initial retinal detachment surgery. Only 3 eyes had previously undergone PPV once with gas tamponade, the remaining 11 (79%) eyes underwent 2 to 5 operations, including SB, PPV with gas tamponade, PPV with SB, PPV with silicone oil tamponade, silicone oil removal, silicone oil removal with silicone oil re-tamponade, phacoemulsification with intraocular lens placement, and posterior scleral reinforcement. Of these eyes, 8 (57%) had undergone silicone oil placement and removal before this surgery and 2 had undergone SB before this surgery. The re-RD involved the fovea in 7 (50%) eyes. There was no break detected in 2 eyes, suspected break in 5 eyes, 1 break in 4 eyes, 2 breaks in 2 eyes, and 3 breaks in 1 eye. The break was superior in 4 eyes, inferior in 2 eyes, both in 1 eye, and suspicious inferior in 5 eyes. The RD involved the inferior retina in 11 eyes. The RD extent was limited to a single quadrant in 4 eyes, two quadrants in 5 eyes, three quadrants in 2 eye, and four quadrants in 3 eyes. The axial length ranged from 22.44 mm to 33.17 mm (26.69 ± 3.47 mm). Six eyes had an axial length more than 26 mm. Seven eyes underwent the procedure with gas injection. The preoperative IOP was 6.2 ~ 23.9 mmHg (11.2 ± 4.4 mmHg) and the BCVA was 2.0 LogMAR~ 0.1 LogMAR (0.9 ± 0.5 LogMAR, Snellen 20/160 ± 20/63). Retinal detachment recurred 2 weeks to 10 years after the last surgery. The duration of symptoms ranged from 1 day to 2 months.

The surgery details are summarized in Table 2. In 2 (case 5 and 9) of the 14 eyes, 360 degree buckling were not applied, the other 12 eyes were given encircling 240 band about 70 mm long overlying the 219 tire which had a different length according to the break/degeneration area. In these 2 cases, retinal tear was clear and PVR was limited to peripheral RD area and the the retinal detachment was fresh. Partial 219 tire was enough to support the retinal breaks and relieve traction. So encircling 240 band were not necessary. Seven eyes underwent the procedure with gas injection at the end of the SB. Subretinal drainage was performed in 9 eyes. None of the eyes had any intraoperative complications.

**Table 2:** Surgery details of all patients in the study

No.	Surgery procedures	SRF drainage	Gas tamponade	Intraoperative complications
1^*^	219 tire+ 240 encircling+cryo+SRF drainage	Y	N	N
2	219 tire+ 240 encircling+cryo+paracentesis of anterior chamber	N	N	N
3	219 tire+ 240 encircling+cryo+SRF drainage+ 0.3 ml SF6 injection	Y	Y 0.3 ml SF6	N
4^*^	219 tire+ 240 encircling+cryo+SRF drainage	Y	N	N
5^&^	posterior scleral enforcement^#^ + 219 tire+cryo+paracentesis of anterior chamber	N	N	N
6	219 tire+ 240 encircling+SRF drainage+paracentesis of anterior chamber	Y	N	N
7^$^	219 tire+ 240 encircling+cryo+paracentesis of anterior chamber+IOL transscleral fixation	N	N	N
8^*^	219 tire revision + 240 encircling+cryo+ 1 ml air injection	N	Y 1 ml air	N
9^&^	219 tire+cryo+SRF drainage+ 0.3 ml C2F6 injection	Y	Y 0.3ml C2F6	N
10^§^	219 tire+ 240 encircling+cryo+ 0.3 ml C2F6 injection	N	Y 0.3ml C2F6	N
11^*^	219 tire+ 240 encircling+cryo+SRF drainage+paracentesis of anterior chamber+ 0.3 ml C2F6 injection	Y	Y 0.3ml C2F6	N
12^*^	219 tire+ 240 encircling+cryo+SRF drainage+air injection	Y	Y 0.8 ml air	N
13	219 tire+ 240 encircling+cryo+SRF drainage	Y	N	N
14	219 tire+ 240 encircling+cryo+SRF drainage+1ml air + 0.18ml C3F8	Y	Y 1 ml air+ 0.18 ml C3F8	N

219 tire: 4.8 mm wide silicon tire with a different length according to the break/degeneration area, 240 encircling: 2.5 mm wide silicon band in about 70 mm long overlying the 219 tire, SRF:subretinal fluid, IOL: intraocular lens

^*^: Cryopexy was applied on suspicious break area in these 5 eyes

&: 360 degree 240 buckling were not applied in case 5 and 9 because retinal tear was clear in these 2 cases and PVR was limited to peripheral RD area and the the retinal detachment was fresh. Partial 219 tire was enough to support the retinal breaks and relieve traction, so encircling 240 band were not necessary. The other 12 eyes were given encircling 240 band about 70 mm long overlying the 219 tire which had a different length according to the break/degeneration area

$: The previously implanted IOL was subluxated and we carried out transscleral fixation of the same IOL

§: A suspicious break was found at 1 o’clock, we did cryopexy.But it did not seem to be clear during cryopexy

#: We carried out posterior scleral reinforcement before scleral buckling because of pathological degenerative changes in the retina including posterior staphyloma, sclera thinning, retinochoroidal atrophy and macular retinoschisis

**Fig. 1:**
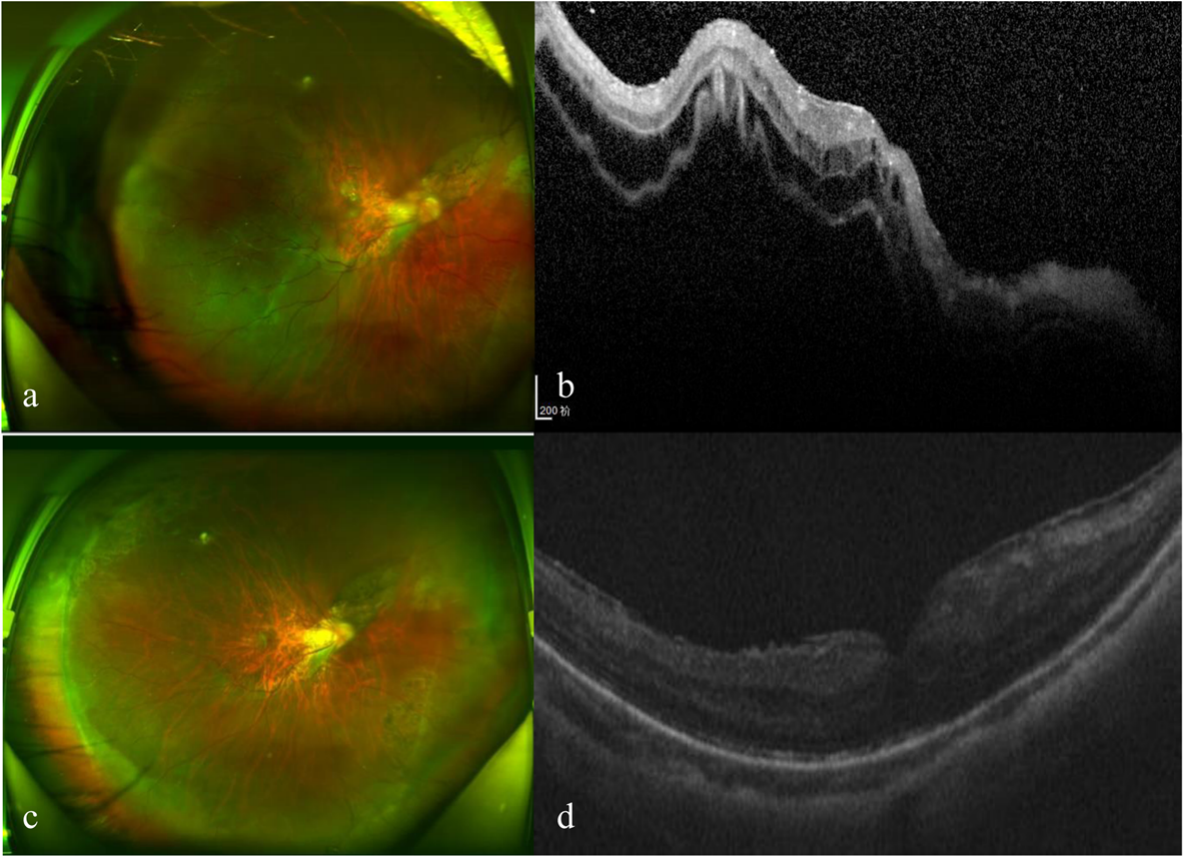
Changes of Case 11 before and after surgery. Fudus photography before SB surgery(**a**). OCT before SB surgery (**b**). Fudus photography 2 months after SB surgery (**c**). OCT 2 months after SB surgery (**d**)

The postoperative follow-up interval ranged from 8 months to 36 months (20 ± 7.9 months). Anatomic success was achieved in all 14 patients one month after the surgery. One eye (Case 11) was observed to have excess subretinal fluid the day after the surgery, which gradually got absorbed one month later (Fig.1). Two eyes (Case 12 and Case 13) had re-RD. Case 12 had some subretinal fluid in the posterior region of the previously compressed temporal retina 4 months later. There was a tiny hole at 10 o’clock. Total retinal reattachment was achieved after vitreous 0.8 ml air injection and laser treatment next day. Case 13 still had some subretinal fluid 1 week later in inferonasal retina and the fluid was absorbed after vitreous 0.6 ml C3F8 injection; however, the retina redetached 1 month later due to unclosure of previous 11 o’clock break and the patient refused retreatment because of poor vision. At the last follow-up, 13 (93%) eyes achieved total retinal attachment and 1 eye had re-RD. The finest BCVA achieved was 20/25 (0.1 LogMAR). There was a statistically significant improvement in the BCVA, from 20/160 ± 20/63 (0.94 ± 0.53 LogMAR) at baseline to 20/80 ± 20/50 (0.57 ± 0.37 LogMAR) at the last visit in the 13 successfully treated eyes (*P* = 0.025). Except for Case 13, (light perception) whose retinal reattachment was unsuccessful, all other eyes maintained or improved their preoperative vision. The postoperative IOP was in the normal range, except in 1 eye (Case 13: 6 mmHg). The mean preoperative IOP of the 13 successful eyes was 12.05 mmHg and mean postoperative IOP was 15.06 mmHg (*P* = 0.115). The follow-up statistics of all patients are summarized in Table 3.

**Table 3:** Follow-up statistics of all patients

No.	Immediate Postop retina status	Retina status on the next day	Preop BCVA in LogMAR (Snellen)	BCVA at the last follow-up in LogMAR (Snellen)	Preop IOP (mmHg)	IOP at the last follow-up (mmHg)	Follow-up time(months)	Retina status at the last follow-up
1	reattached	reattached	1.40 (20/500)	1.30 (20/400)	8.0	15.4	36	attached
2	some SRF	reattached	1.00 (20/200)	0.50 (20/63)	14.0	10.8	30	attached
3	reattached	reattached	0.20 (20/32)	0.20 (20/32)	8.0	Tn	30	attached
4	reattached	reattached	0.40 (20/50)	0.10 (20/25)	10.0	Tn	23	attached
5	some SRF	reattached	0.10 (20/25)	0.10 (20/25)	10.0	15.5	30	attached
6	reattached	reattached	1.40 (20/500)	0.40 (20/50)	10.0	13.0	18	attached
7	some SRF	reattached	2.00 (20/2000)	0.20 (20/32)	8.0	13.0	15	attached
8	some SRF	reattached	0.70 (20/100)	0.70 (20/100)	13.0	17.0	21	attached
9	reattached	reattached	0.80 (20/125)	0.80 (20/125)	15.0	20.0	19	attached
10	some SRF	reattached	0.90 (20/160)	0.90 (20/160)	23.9	13.0	18	attached
11	reattached	detached	1.00 (20/200)	0.50 (20/63)	11.0	13.0	18	attached
12	reattached	reattached	1.00 (20/200)	0.80 (20/125)	10.8	15.0	8	attached
13	reattached	detached	1.00 (20/200)	2.30 (light perception)	6.2	6.0	16	detached
14	reattached	reattached	1.30 (20/400)	0.90 (20/160)	8.8	20.0	8	attached

Postop: postoperative, Preop: preoperative, SRF: subretinal fluid

## Discussion

Causes of retinal re-detachment after PPV include the following: PVR; factors associated with breaks such as ineffective closure of preexisting breaks, large breaks, opening of old breaks formation of new breaks, reopened macular hole, and progressive vitreoretinal traction; incomplete removal of the vitreous base and shaving; inadequate retinal tamponade not adhering to the strict continuous posture; and perisilicone proliferation [1, 9]. The most common cause of recurrent RD is PVR [12]. Contraction of PVR may cause retinal foreshortening, which can exert anteroposterior, perpendicular, and/or circumferential traction on the retina, particularly near the vitreous base [3]. Furthermore, foreshortening of the retina may prevent retinal reattachment. SB can relax the tractional forces on the retina, thus reattaching the retina effectively. All our 14 cases had PVR, and at the last follow-up, 13 of the 14 patients achieved anatomical success, supporting the importance of relaxing the tractional forces when reattaching the retina.

In a review, Edwin and Robert analyzed the continued role of SB in the vitrectomy era. They found that proficient skill and practice are required to correctly place the SB elements with the desired indentation to support the retinal tears and to drain the subretinal fluid without complications [13]. In our research, the RDs were not primary retinal detachment, but re-RD with PVR. Nevertheless, we had a high success rate. This could be attributed to the surgeon’s experience and careful patient selection. From our experiennce, we propose that encircling buckling should be applied in the following cases:(1) The retina is rigid;(2) High myopia with long axial length;(3) The retinal break is not clear;(4) After multiple PPV and/or after silicon oil removal. We suggest using simple scleral tire in the following cases:(1) Fresh retinal detachment without PVR;(2) The retinal tear is clear with localized retinal detachment;(3) The location of retinal tear is between superior 8 to 4 o’clock. Generally, the criteria for case selection are as follows: (1) The (probable) retinal tear is on the periphery; (2) There is no obvious proliferative traction in the posterior retina; and (3) The height of RD is low. Patients with funnel-shaped RD cannot undergo this surgery.

RD in post-vitrectomy eyes can progress rapidly, which can easily lead to PVR. Furthermore, if another PPV is performed to attach the retina, silicone oil may be used, often causing the eye to become oil dependent. However, the presence of silicone oil does not guarantee retinal reattachment. The recurrence rate of RD in silicone-oil filled eyes is of 22% [14]. The postoperative complications associated with using silicone oil occur both in the anterior and posterior segments, including keratopathy, cataract, glaucoma, silicone oil toxicity in the retina [14, 15]. The longer the silicone oil remains in the eye, the more complications arise. Some authors have reported extraocular extension of silicone oil into the brain 15 months after silicone oil tamponade in the eye [16, 17]. The risk of re-RD is 34% after removal of silicone oil [6]. It is thus important to study the outcomes of other interventions to understand whether performing repeated vitrectomy on patients with a history of failed surgeries is worthwhile. Our research on re-RD in post-vitrectomy eyes with prior RRD demonstrated the benefits of SB.

Twelve out of 14 patients (86%) achieved anatomical success after one operation and 13 out of 14 patients (93%) achieved final anatomical success, which is comparable with previous re-vitrectomy + retinectomy + gas or silicone oil tamponade reports that showed a reattachment rate of 60 to 90% [2, 3, 8, 12]. The inability to detect retinal breaks in a RRD has been reported to be associated with a poor prognosis [18]. In our patients, no clear breaks were detected in 7 eyes preoperatively. Six of the 7 eyes were pseudophakic and the remaining one eye was aphakic. Five of the 7 eyes had suspicious holes and 2 eyes had no visible break during the operation; nevertheless, retinal reattachment was successful in all these cases, which indicated that carefully selected re-RD as mentioned above without detecting a break is not a contraindication for 360 degree SB. As for eyes without visible breaks, SRF drainage or gas injection or both should be given for the technique to work. In eyes with definite breaks, closure of the break should be ensured at the end of the surgery by indentation of scleral buckling whether it’s combined with SRF drainage or not. Not all of our cases were given SRF drainage because some eyes had only a small amount of fluid, which can make performing an operation difficult. On the other hand, the neuroretina aroud the break was close to the retinal pigment epithelium upon indentation in these cases. Foster etal [19] considered that scleral buckling can induce drainage of subretinal fluid to the vitreous cavity and is thus beneficial to retinal reattachment. The reasons why breaks cannot be detected are as follows: the IOL occludes some small breaks, the break is too small to be found, the RD is too high and the break is hidden in the PVR, or there is no break present.

In 2 eyes of our patients, recurrence of retinal detachment appeared after sceral buckling. We gave them rescue pneumatic retinopexy. Reattachment in case 12 was successful, but the procedure failed in case 13. We concluded that a newly emerging diffuse posterior traction in case 13 impeded the reattachment, while the encircling band in case 12 effectively released previous PVR. That is also why we did not carry out simple pneumatic retinopexy /fluid gas exchange with cryo at first. The retinal detachment in case 12 can be attributed to the formation of a new break.

Despite having undergone a mean of 3.29 surgeries at the last visit, 5 (36%) of our 14 patients still had a final BCVA≥0.4 LogMAR (Snellen 20/50), which is also promising compared with previous reports [9, 12, 20]. Macular involvement with RD is a known risk factor for a limited visual outcome [5, 18]. In our cases, 7 patients had macula-on retinal detachment. One eye maintained the preoperative vision, 5 eyes had improved vision, with the best BCVA reaching 0.1 LogMAR (Snellen 20/25), and one eye had decreased vision because of failure to reattach the retina.

The advantages of SB for treating recurrent RD after PPV are mainly as follows: (1) It alleviates damage to the eye using minimal surgery compared with PPV. (2) It does not interfere with the intraocular tissue (SB is an external operation), which can reduce irritation to the posterior retina, thus may protecting the macula to some extent [2, 21]. (3) Patients need not remain in a prone position or only for a short time after operation if it is combined with gas injection, thus relieving the patient’s discomfort. (4) Repeated PPV procedures are inherently more expensive than SB, and it increases difficulty in operating and usually leads to silicone oil dependent eyes, causing both economical and emotional burden. Moreover, the presence of silicone oil does not guarantee correct retinal positioning, with an RD recurrence rate of 22% [14].

The disadvantages of SB for treating recurrent RD after PPV are mainly as follows: (1) SB greatly differs from PPV, and there is a significant learning curve. Experience with many cases is required to accurately select the most effective elements [13]. Therefore, both proficiency in indirect ophthalmoscopy and caution when performing SB procedures are necessary. Experience and correct technique to treat complications are necessary. (2) Ocular surface inflammation is more severe after SB, but it quickly recovers. (3) It can cause anterior and posterior segment ischemia [22]. Though the chances are low, irreversible visual impairment can be damaging. (4) The buckle material can cause some changes in the eyeball structure, inducing myopia and astigmatism [23]. (5) It can induce ocular motility disorders (transient or permanent) and squint/strabismus [22].

Improvements in the instruments used for vitrectomy and the introduction of 25-gauge vitrectomy has minimized the wound and decreased the necessity of suturing. Hence, it may have increased the surgeons’ preference to perform vitrectomy over SB [23]. Nevertheless, multiple PPVs may not always be the best strategy for patients, and SB may be more beneficial.

There are some limitations to our study. Firstly, the sample size was small. Furthermore, at the end of the SB surgery, gas (air or C2F6) was injected in some eyes, which may have affected the evaluation of SB surgery alone. Nevertheless, the preliminary results of our study are promising, and clinicians should consider whether SB is feasible instead of repeated vitrectomy for patients with a history of failed surgeries. However, further prospective clinical trials with a larger number of cases are necessary for a full evaluation of the effect of SB, and for determining the optimal surgical procedure for the treatment of re-RD after PPV.

## Conclusions

To the best of our knowledge, this is the first report to demonstrate the effectiveness of SB for re-RD in specific eyes after PPV. Scleral buckling can be effective for re-RD after PPV in specific cases. In some cases, it may be more beneficial than repeated PPVs.

## Data Availability

The datasets generated during and analyzed during the current study are not publicly available due to patient privacy and intellectual property protection, but are available from the corresponding author on reasonable request.

## Abbreviations

SB: Scleral buckling
re-RD: recurrent retinal detachment
PPV: Pars plana vitrectomy
BCVA: Best-corrected visual acuity
RRD: Rhegmatogenous retinal detachment
MHRD: Macular hole retinal detachment
logMAR: logarithm of the minimum angle of resolution
SRF: Subretinal fluid
